# Extracellular and Luminal pH Regulation by Vacuolar H^+^-ATPase Isoform Expression and Targeting to the Plasma Membrane and Endosomes*

**DOI:** 10.1074/jbc.M116.723395

**Published:** 2016-02-24

**Authors:** Gina A. Smith, Gareth J. Howell, Clair Phillips, Stephen P. Muench, Sreenivasan Ponnambalam, Michael A. Harrison

**Affiliations:** From the ‡ Endothelial Cell Biology Unit, School of Molecular and Cellular Biology and; § School of Biomedical Sciences, Faculty of Biological Sciences, University of Leeds, Leeds LS2 9JT, United Kingdom

**Keywords:** endocytosis, membrane trafficking, receptor internalization, receptor recycling, vacuolar ATPase

## Abstract

Plasma membrane vacuolar H^+^-ATPase (V-ATPase) activity of tumor cells is a major factor in control of cytoplasmic and extracellular pH and metastatic potential, but the isoforms involved and the factors governing plasma membrane recruitment remain uncertain. Here, we examined expression, distribution, and activity of V-ATPase isoforms in invasive prostate adenocarcinoma (PC-3) cells. Isoforms 1 and 3 were the most highly expressed forms of membrane subunit *a*, with *a*_1_ and *a*_3_ the dominant plasma membrane isoforms. Correlation between plasma membrane V-ATPase activity and invasiveness was limited, but RNAi knockdown of either *a* isoform did slow cell proliferation and inhibit invasion *in vitro*. Isoform *a*_1_ was recruited to the cell surface from the early endosome-recycling complex pathway, its knockdown arresting transferrin receptor recycling. Isoform *a*_3_ was associated with the late endosomal/lysosomal compartment. Both *a* isoforms associated with accessory protein Ac45, knockdown of which stalled transit of *a*_1_ and transferrin-transferrin receptor, decreased proton efflux, and reduced cell growth and invasiveness; this latter effect was at least partly due to decreased delivery of the membrane-bound matrix metalloproteinase MMP-14 to the plasma membrane. These data indicate that in prostatic carcinoma cells, *a*_1_ and *a*_3_ isoform populations predominate in different compartments where they maintain different luminal pH. Ac45 plays a central role in navigating the V-ATPase to the plasma membrane, and hence it is an important factor in expression of the invasive phenotype.

## Introduction

The vacuolar H^+^-ATPase has emerged as a significant contributor to net proton efflux from tumor cells (1–4). This 1-MDa membrane complex, comprising the soluble catalytic V_1_ domain coupled to an integral membrane domain V_o_, uses the free energy of cytosolic ATP hydrolysis to pump protons across membrane bilayers (5, 6). Also a ubiquitous component of endomembranes, the V-ATPase acidifies compartments involved in all stages of vesicular transport (6). Plasma membrane V-ATPases are not unique to tumor cells and also play crucial roles in acid efflux from osteoclasts, epididymal clear cells, and renal intercalated cells (7, 8).

The ATP-hydrolyzing V_1_ domain contains subunits A–H in a stoichiometry (AB)_3_CDE_3_FG_3_H. V_o_ translocates protons across the membrane and consists of *a*, *d,* and *e* subunits linked to a 6–10-member ring of *c* subunits. Operating as a rotary motor, proton translocation is postulated to take place at the dynamic interface of the *a* subunit and *c*-ring in the membrane (see Ref. 5 and references therein). In higher eukaryotes, most subunits occur as multiple isoforms (9, 10). For many, a common ubiquitous form predominates with others expressed at high levels in particular tissues. The B_1_ subunit (ATP6V1B1), for example, is highly expressed in kidney, with naturally occurring mutations causing distal renal tubular acidosis, whereas B_2_ is ubiquitously expressed (11). Vertebrates express subunit *a* isoforms 1–4 (plus additional splice variants) that have 47–61% sequence identity (12, 13). Although individual subunit *a* isoforms are particularly highly expressed in some cells, multiple forms are generally present and targeted to specific compartments (14, 15). Targeting may be a function of the cytoplasmic N-terminal domain of the protein, as it is in the budding yeast *Saccharomyces cerevisiae* (16). The *a*_1_ form (ATP6V0A1) appears ubiquitous, with some variants particularly abundant in neuronal tissue (17), whereas *a*_3_ (ATP6V0A3) is highly expressed in osteoclasts and macrophages (14, 18). The predominant species in cells of the kidney and male reproductive tract is *a*_4_ (19, 20). The *a*_2_ form has been detected in early endosomes (21) and in the Golgi complex (14, 22, 23), with mutations causing the glycosylation defects and aberrant trafficking that are characteristic of the disease cutis laxa (22). The *a*_3_ form is found in late endosomes/lysosomes of cells of monocytic lineage from where it is recruited to the ruffled border zone of the plasma membrane of active osteoclasts or to the maturing phagosome of macrophages (14, 18). In pancreatic β-cells, *a*_3_ is targeted to insulin-secreting granules (24), although the *a*_1_ form has been identified in secretory vesicles in neuroendocrine PC12 cells (23). The *a*_1_ isoform is also found in synaptic vesicles of neuronal cells (17), performing the following two distinct roles: proton pumping to energize neurotransmitter loading into vesicles, and Ca^2+^-regulated vesicle fusion with the plasma membrane involving V_o_ interaction with the R-SNARE synaptobrevin (VAMP2) (25, 26). Consequently, V_o_ containing subunit *a*_1_ is likely to appear at least transiently at the plasma membrane of neuronal cells (17), although not necessarily actively coupled to V_1_. The *a*_1_, *a*_3_, and *a*_4_ forms are also featured at the plasma membrane of kidney proximal tubule cells, in which *a*_2_ appears to be localized to early endosomes (21). In renal intercalated cells and epididymal clear cells, *a*_4_ is targeted to the apical membrane (27). Currently, little is known about either the functional differences between subunit isoforms or the rules governing the interactions they make, but it is feasible that eukaryotic cells could assemble a wide variety of V-ATPase complexes with potentially different functional properties, locations, and inhibitor sensitivity.

Plasma membrane V-ATPase activity is linked to growth and invasiveness of a number of neoplastic cell types, including breast (1, 3, 28, 29) and prostate (30, 31). Expression of the subunit *a*_3_ isoform is higher in invasive melanoma cells compared with non-invasive controls (32), and the same subunit is also implicated in invasiveness of breast cancer cells (33). The *a*_4_ subunit is highly expressed in some gliomas (34). V-ATPase activity is proposed to contribute to tumor cell invasiveness and growth via the following three mechanisms: first, maintaining the acidic extracellular pH (pH_ex_) promotes activity of cathepsins and activation of matrix metalloproteases (30, 32, 35, 36) that can remodel the ECM^6^ to facilitate tumor cell extravasation and invasion of adjacent tissues; second, by contributing to the stability of the abnormally alkaline cytosolic pH (pH_cyt_) of tumor cells (37), the V-ATPase helps the cell evade acidosis-induced apoptosis (38, 39); and third, V-ATPase activity facilitates sequestration of some chemotherapeutic drugs in intracellular compartments leading to drug resistance (29, 40, 41). The sensitivity of tumor cells to loss of V-ATPase function has fueled interest in the enzyme as a therapeutic target in cancer (42, 43), but the ubiquitous nature of the enzyme makes specific targeting of cancer cells problematic. V_o_ subunit *c* binds bafilomycin and other lipid-soluble inhibitors (44, 45), but a role for subunit *a* in inhibitor binding is also likely (46, 47), opening the possibility of discriminatory isoform-specific inhibitors. To support such development, more information is required about the differential expression, relative levels of activities, and functional roles of the different isoforms in cancer cells.

In this study, we have examined the expression of subunit *a* isoforms in prostatic carcinoma cells and examined their relative contributions to proton efflux activity across the plasma membrane. Using RNAi, we looked at the function of different isoforms in endocytotic processes such as plasma membrane receptor recycling. The accessory subunit Ac45 has been proposed to be a primary factor in V-ATPase relocation to the plasma membrane (48, 49) and in Ca^2+^-regulated exocytosis (50). Here, we investigated the association of this polypeptide with different subunit *a* isoforms and the consequences of its depletion on V-ATPase localization and function in prostate carcinoma cells.

## Experimental Procedures

### 

#### 

##### Cell Culture

PC-3 (derived from a grade IV prostatic adenocarcinoma bone metastasis) and LNCaP (lymph node metastasis of prostatic carcinoma) epithelium-like cell lines obtained from ECACC were cultured in Ham's F-12 and RPMI 1640 media, respectively, supplemented with 7% fetal bovine serum and 2 mm glutamine. Cultures were incubated at 37 °C under 5% CO_2_. For transfer to microphysiometry and invasion assay supports, the adherent cells were released by treatment with Accutase (PAA Laboratories). Two cultures of PC-3 cells used at different stages in this study were both validated by STR profiling (Public Health England Cell Line Authentication Service, Porton Down, UK).

##### RNAi Treatment

Cells cultured in 6-well plates were treated with 19-mer siRNAs targeted against Ac45 (Thermo Scientific-Dharmacon SMARTpool M-021378-00, 25 nm), ATP6V0A1 (Thermo Scientific-Dharmacon SMARTpool M-017618-00, 100 nm), ATP6V0A3 (Thermo Scientific-Dharmacon SMARTpool M-012198-00, 100 nm), and ATP6V1A1 (Thermo Scientific Dharmacon SMARTpool L-017590-01, 50 nm). A control siRNA (Thermo Scientific-Dharmacon non-targeting pool D-001810-10) was also used at 100 nm. The four constituent siRNAs within each pool were also tested individually for effects on expression and for phenotypic effects. As an additional negative control, a representative siRNA from each pool was tested after custom synthesis (Thermo Scientific-Dharmacon) to include nucleotide changes as underlined: *a*_1_, caacaucucaguaaugcua; *a*_3_, cccucgcgcagcacaagua; Ac45, ccaccugucaacguaguuu).

The siRNAs were incubated with DharmaFECT-2 transfection reagent (Thermo Scientific) in serum-free medium for 45 min at room temperature before mixing 1:4 with complete medium (7% fetal bovine serum). Cells were treated with the siRNA mixture for 24 h before replacement with fresh complete medium. Where necessary for certain assays (invasion, microphysiometry), cells were transferred after this time and used within the subsequent 24 h.

##### Microphysiometry

Measurements of extracellular acidification rate (ECAR) were performed using a Cytosensor microphysiometer (Molecular Devices). Adherent PC-3 cells (10^5^ cells per cup) were seeded directly into the microphysiometer capsule cups and cultured for 48 h as above to allow an adherent monolayer to form. Weakly adherent LNCaP cells required embedding in agarose to prevent detachment from the capsule membrane. Cups were placed in the sensor chamber containing perfusion buffer (nominally bicarbonate-free Hanks' basal salt solution (HBSS) without HEPES, pH 7.4). For sodium-free HBSS, NaCl was replaced with 138 mm choline to maintain osmolarity. The flow chamber formed between cell monolayer and insert (effective volume 2.6 μl) was circulated with perfusion buffer at 37 °C at a rate of 100 μl min^−1^. A pump cycle of 40 s off and 80 s on was used. Typically, cells reached a steady state ECAR after 30–40 min. Rates of acid accumulation are computed by the Cytosensor software as the least squares fit to the raw voltage time trace recorded when the pump is off, and the derived data are expressed as the change in ECAR as a function of time. Buffering capacity of the perfusion buffer, equilibrated with atmospheric CO_2_ at 37 °C, was determined by titrating change in pH with increasing concentrations of HCl. Titration curves were linear across the pH range spanned by the experiments. Rates of proton efflux (*J*_H_^+^, in nm H^+^ min^−1^) were calculated by first converting ECAR into a rate of change of pH (using the Nernst equation) and then calculating the increase in H^+^ concentration assuming a starting concentration of 40 nm, pH 7.4. Because the buffering capacity of the perfusion medium was negligible, *J*_H_^+^ was not corrected for any buffering effects. At 37 °C, a change in pH_ex_ of 1 pH unit min^−1^ is equivalent to a rate of change in potential of 61 mV s^−1^.

Inhibitors were perfused into the assay chamber via electronically switchable valves. Bafilomycin A_1_, concanamycin A, and ethylisopropylamiloride (Sigma) were introduced from freshly made stock solutions in DMSO. This vehicle had no measurable effects on ECAR.

##### Cell Proliferation Assay

Cells were plated at a density of 1 × 10^4^ cells/well in a 96-well plate (CellCarrier-96, PerkinElmer Life Sciences) and allowed to adhere for 4 h. Cells were then transfected with siRNAs as above. After culture for 48 h, live cell numbers (relative to control) were determined by uptake of calcein acetoxymethyl ester (calcein-AM; 2.5 μm, 60 min incubation at 37 °C in HBSS buffer). Fluorescence was measured in a FlexStation 3 (Molecular Devices) with excitation at 490 nm and emission at 520 nm. Numbers of dead cells (as a percentage relative to controls) were determined fluorometrically by propidium iodide uptake (excitation at 535 nm and emission at 617 nm).

##### Invasion Assays

Cell migration through basement membrane extract was assayed using Cultrex basement membrane extract cell invasion assay in a 24- or 96-well plate format (Trevigen Inc., Gaithersburg, MD). Cells were starved overnight in serum-free medium, detached by treatment with Accutase, and resuspended in serum-free medium to give 10^6^ live cells ml^−1^. In the final assay setup, each upper well of the modified Boyden chamber contained 5 × 10^4^ cells (24-well format) or 0.5 × 10^4^ cells (96-well format). To test the effects of concanamycin, the inhibitor was added to an aliquot of the cell suspension to give a final concentration of 1 μm in the upper chamber (0.5% v/v DMSO, also included in control wells). Soluble recombinant receptor activator of nuclear factor κB ligand (PeproTech, London, UK) was used at 10 μg ml^−1^. As chemoattractant, the bottom well of each chamber contained medium supplemented with 10% FBS. Plates were incubated at 37 °C (5% v/v CO_2_) for 24 h, after which time any cells that had passed through the basement membrane extract/polycarbonate membrane were detached. Cells were quantified by incubating with calcein-AM and measuring the internalized fluorescence signal in a plate reader accessory attached to a Cary Eclipse scanning fluorimeter or FlexStation 3 (excitation at 485 nm and emission at 520 nm).

##### Immunoprecipitation and Immunoblotting

Antibodies used in this study were as follows: mouse anti-subunit A (H-00000523-M02; Abnova Inc., Taiwan) recognizing residues 508–618 of human ATP6V1A; rabbit anti-*a*_1_ (sc-28801, Santa Cruz Biotechnology Inc., Dallas, TX) recognizing residues 71–210 of human ATP6V0A1; rabbit anti-*a*_1_ (AP5109A, Generon, UK) recognizing residues 44–71 of human ATP6V0A1; mouse anti-*a*_2_ (H00023545-A01, Abnova Inc.) recognizing residues 198–304 of human ATP6V0A2; rabbit anti-*a*_3_ made in-house against a peptide comprising residues 689–707 of human ATP6V0A3; rabbit anti-*a*_4_ (ab55827, Abcam, UK) recognizing the C-terminal residues of human ATP6V0A4; mouse anti-Ac45 (H00000537-M01, Abnova Inc.) recognizing residues 51–151 of human ATP6AP1; goat anti-ATP6V1F (sc-21220, Santa Cruz Biotechnology, Inc.) recognizing a C-terminal peptide of V_1_ subunit F; rabbit anti-MMP-14 (AB8102, Millipore (UK) Ltd., Watford, UK), recognizing catalytic domain residues 91–246; mouse anti-CD71 (transferrin receptor; sc-65887, Santa Cruz Biotechnology, Inc.); mouse anti-LAMP-1 (MA1–164, Thermo Fisher); sheep anti-TGN46 (51); and mouse anti-tubulin (T6199 (clone DM1A), Sigma). Whole cell lysates were prepared by solubilization in RIPA buffer (20 mm Tris-HCl, 150 mm NaCl, 1 mm Na-EDTA, 1 mm EGTA, 1% w/v Nonidet P-40, 0.5% sodium deoxycholate, protease inhibitor mixture), centrifugation at 13,000 rpm in a microcentrifuge at 4 °C followed by heating to 80 °C for 10 min. The protein concentration was determined using the BCA method, and the concentration was adjusted to 1 mg ml^−1^ with RIPA buffer. For immunoprecipitation, cells cultured in 6-well plates were washed twice with PBS containing Mg^2+^ and Ca^2+^ (PBS-Mg/Ca) before extraction into RIPA buffer by incubation on ice for 20 min. Cell lysates were centrifuged at 100,000 × *g* for 20 min at 4 °C in a Beckman Optima ultracentrifuge to remove insoluble material. The protein concentration of the cell lysates was assayed, and the volume was adjusted with RIPA buffer to give 1 mg ml^−1^ protein. For immunoprecipitation, 50 μl of rabbit anti-*a*_3_ antiserum or 2 μg of anti-*a*_1_ rabbit IgG were added to 0.4 ml of cell lysate, which was then incubated at 4 °C for 6 h on a shaking platform. Pre-immune rabbit serum was used as a control. The ImmunoCruz IP/WB Optima B system (Santa Cruz Biotechnology) was used to precipitate antibody-bound proteins where subsequent detection of co-precipitated Ac45 and A_1_ was performed with mouse antibodies. For detection of *a*_1_ and *a*_3_, antibody-protein complexes were precipitated by incubation with protein-A/G agarose (Thermo Scientific) for 2 h at 4 °C.

Bead-antibody-protein complexes were recovered by low speed centrifugation, and the pelleted beads were washed three times with RIPA buffer. These were then incubated with SDS-PAGE sample buffer (NuPAGE LDS sample buffer; Life Technologies, Inc.) and heated to 90 °C for 10 min before separation on 4–12 or 10% acrylamide NuPAGE gels (Life Technologies, Inc.). For immunoblotting, protein gels were blotted onto polyvinylidene difluoride membrane and probed with HRP-conjugated goat anti-mouse or anti-rabbit IgG as appropriate. Chemiluminescent signal was captured using a LAS-3000 imaging work station (Fuji Corp., Japan). Exposure times were typically <2 min, with the exception of the *a*_4_ antibody where the detectable signal necessitated 20 min of exposure (see Fig. 2, *B* and *E*). The signal from all immunoblots was within the dynamic range of the imaging workstation.

##### Extraction of Biotinylated Cell Surface Proteins

Cells grown on 6-well plates were washed three times with PBS-Mg/Ca and then incubated on ice on a rocking table for 60 min with a solution of 0.2 mg ml^−1^ EZ-Link sulfo-NHS-SS-biotin (Thermo Scientific) (from fresh 20 mg ml^−1^ stock in DMSO) in PBS-Mg/Ca. Cells were then incubated for 20 min with ice-cold PBS-Mg/Ca containing 100 mm lysine. After aspirating the lysine solution, cells were extracted into RIPA buffer and incubated on ice for 30 min.

To extract a total membrane fraction, cell monolayers from two 175-cm^2^ tissue culture flasks were recovered by scraping into PBS (supplemented with protease inhibitors), sonicated on ice for 60 s, and centrifuged at 10,000 × *g* for 20 min at 4 °C. The supernatant was then centrifuged at 100,000 × *g* for 1 h at 4 °C. The pellet corresponding to a total membrane fraction was resuspended in PBS containing 0.2 mg/ml EZ-Link sulfo-NHS-SS-biotin and incubated on ice for 60 min before addition of lysine (100 mm). The membranes were then recovered by centrifugation at 100,000 × *g* as above, washed with PBS, and finally resuspended in RIPA buffer before incubation on ice for 30 min.

For recovery of biotinylated proteins, RIPA buffer extracts were centrifuged at 100,000 × *g* for 20 min and then added to magnetic StrepTactin beads (Qiagen) pre-equilibrated with RIPA buffer. Beads were agitated for 60 min at 4 °C, then recovered using a magnet, followed by two washes with RIPA, one wash with 0.2× RIPA (RIPA buffer with 0.2% w/v Nonidet P-40, 0.1% w/v sodium deoxycholate). The recovered beads were incubated at 90 °C with SDS-PAGE sample buffer, separated by SDS-PAGE, and probed by immunoblotting as above.

To monitor recycling of plasma membrane proteins, cells were cultured, washed, and labeled with sulfo-NHS-SS-biotin as above. After biotinylation and quenching with lysine, control cells (representing a maximally surface-labeled fraction) were removed into RIPA buffer. Internalization of biotinylated surface proteins was initiated by replacing cold PBS-Mg/Ca with HBSS warmed to 37 °C. At 5–120-min time points, residual surface biotin was removed by incubation with 100 mm sodium 2-mercaptoethanesulfonate (MESNa) (in 100 mm NaCl, 1 mm EDTA, 0.2% w/v BSA, 50 mm Tris-HCl, pH 8.6) on ice for 15 min with gentle rocking. MESNa was then quenched by washing with ice-cold 100 mm iodoacetamide in PBS-Mg/Ca. Cells were suspended in ice-cold PBS by aspiration and counted in a hemocytometer. Cells were then recovered by centrifugation (5000 × *g*, 10 min, 4 °C) and extracted into RIPA buffer with the volume adjusted according to cell count to give an equivalent volume per cell. This suspension was incubated on ice for 30 min. Lysates were centrifuged at 100,000 × *g* for 20 min (4 °C), and biotinylated proteins within the supernatants were bound to magnetic StrepTactin beads, washed, and analyzed by SDS-PAGE/immunoblotting as above.

##### Vesicular pH Assay

Cells were grown in 24-well plates and incubated with FITC-dextran ((Sigma) 0.5 ml, 0.2 mg ml^−1^) in PBS (with Mg^2+^ and Ca^2+^) for 10 min at 37 °C. Total uptake was determined by washing cells three times with PBS-Mg/Ca at 4 °C, before harvesting by aspiration. Cells were resuspended in PBS with 100 mm KCl and 10 μm nigericin and placed in a 96-well fluorimeter plate, and the fluorescence was determined in a Cary Eclipse fluorimeter with excitation at 490 nm and emission across the 510–560-nm range. To determine vesicular pH, after washing with PBS-Ca/Mg, cells were incubated with HBSS at 37 °C. At time intervals of 5–60 min, cells were recovered into PBS and placed in 96-well fluorimeter plate, and the excitation spectrum in the 400–500 nm range was obtained for emission at 520 nm (3 scan average, excitation slit 2.5 nm, emission slit 5 nm, 1200 nm min^−1^ scan speed). The time-dependent change in the ratio of excitation at 492 nm to that at 442 nm for 520-nm emission was then calculated. For calibration, cells were resuspended in PBS, 100 mm KCl, 10 μg ml^−1^ nigericin with 25 mm MES, 25 mm MOPS with the pH adjusted to 5, 6, 7, or 8 with KOH.

##### Fluorescence Microscopy

For fluorescence microscopy, cells were grown on polylysine-treated coverslips until 70–80% confluent. Coverslips were washed three times with PBS-Ca/Mg before fixing with 4% w/v paraformaldehyde in PBS-Mg/Ca (10 min, room temperature). Paraformaldehyde was quenched by adding NH_4_Cl to 50 mm in PBS (10 min at room temperature), followed by washing with PBS. Cells were permeabilized with 1% w/v Triton X-100, 1% w/v fish skin gelatin in PBS for 10 min at room temperature, then incubated with 1:250 dilution of anti-*a*_3_ polyclonal antiserum or with 5 μg ml^−1^ purified antibodies for 1 h at room temperature. For MMP-14 labeling, Triton X-100 was omitted. Cells were then washed two times with PBS (20 min each) and then with 1:250 dilution of normal goat serum in PBS. After briefly washing with PBS, Alexa-488-labeled goat anti-rabbit or Texas Red-labeled goat anti-mouse antibodies (Life Technologies, Inc.) were added for 1 h at room temperature, each also containing Hoechst 33258 (1 μg ml^−1^) for nuclear staining. After washing three times with PBS (20 min each), coverslips were gently blotted and mounted onto slides with Fluoromount G (Southern Biotech), then gently sealed with acrylic, and stored in dark at 4 °C. For controls, primary antibodies were replaced with pre-immune rabbit serum or with purified rabbit or mouse control IgG.

Images were acquired using a DeltaVision deconvolution microscope controlled by the SoftWorx suite of programs at ×60 magnification. After locating upper and lower sections of each cell, image stacks in the z-plane were collected typically as 2–3-μm sections at three excitation/emission settings (for Hoechst 33258, Alexa-488, and Texas Red/Alexa-594). Image acquisition settings (exposure time, filter settings, pixel binning, and excitation power) were kept constant for all images to allow valid comparison and quantitation. Image deconvolution was performed using the corresponding function in SoftWorx. Images of anti-MMP-14 antibody-labeled cells were collected on an EVOS FI inverted digital microscope (Life Technologies, Inc., Paisley, UK).

Pixel coincidence in double-labeled images was quantified using the “co-localization” function of the image analysis software Imaris (Bitplane AG, Zurich, Switzerland). For each channel, background fluorescence was set at 10% of the total signal. Nonspecific binding of control mouse and rabbit IgG and rabbit pre-immune serum were tested but gave negligible signal using the image acquisition settings used consistently for collecting data. Texas Red anti-mouse and Alexa-488-rabbit secondary antibodies were tested, respectively, for their species cross-reactivity to the rabbit (*a*_1_ and *a*_3_) and mouse (Ac45/TfR/LAMP-1/A) primary antibodies, but again the negligible signal was detected.

##### Transferrin Uptake Assay

Cells were grown on glass coverslips as above and incubated in serum-free medium for 2 h. Cells were then incubated with Alexa-594-conjugated human transferrin (Molecular Probes, Life Technologies, Inc.) at 25 μg ml^−1^ on ice for 30 min. As a control, a set of coverslips was also pre-treated with the endocytosis inhibitor Dynasore (80 μm) for 30 min before exposure to transferrin. Cells were then washed twice with ice-cold PBS-Mg/Ca to remove excess transferrin. “Time 0” cells were immediately fixed for microscopy with paraformaldehyde as above. Transferrin transport was initiated by addition of HBSS warmed to 37 °C, and the cells were moved to an incubator. Coverslips were removed at time points 30–90 min, washed briefly with PBS-Mg/Ca, and processed for microscopy as above, with additional staining for the *a*_1_ subunit and with Hoechst 33258. Images were captured using the DeltaVision microscope with fixed acquisition parameters throughout. For each time point, images of 18–30 cells were collected. Quantitation of Alexa-594-tranferrin fluorescence was performed using ImageJ software (rsb.info.nih.gov).

##### Gene Expression Profiling

RNA was isolated from PC-3 cells using RNeasy micro-extraction kit (Qiagen, Germany) and quantified, and the purity was determined by measuring absorbance at 280, 260, and 230 nm using an ND-1000 nanodrop spectrophotometer (LabTech International, UK). After reverse transcription, cDNA was subjected to microarray analysis using the Human Genome U133 Plus 2.0 array (Affymetrix, High Wycombe, UK). Gene expression levels were analyzed using Microarray Suites software. Sequences used in the design of this array were selected from the GenBank^TM^, dbEST, and RefSeq databases.

## Results

### 

#### 

##### V-ATPase Activity at the Plasma Membrane

**FIGURE 1.**
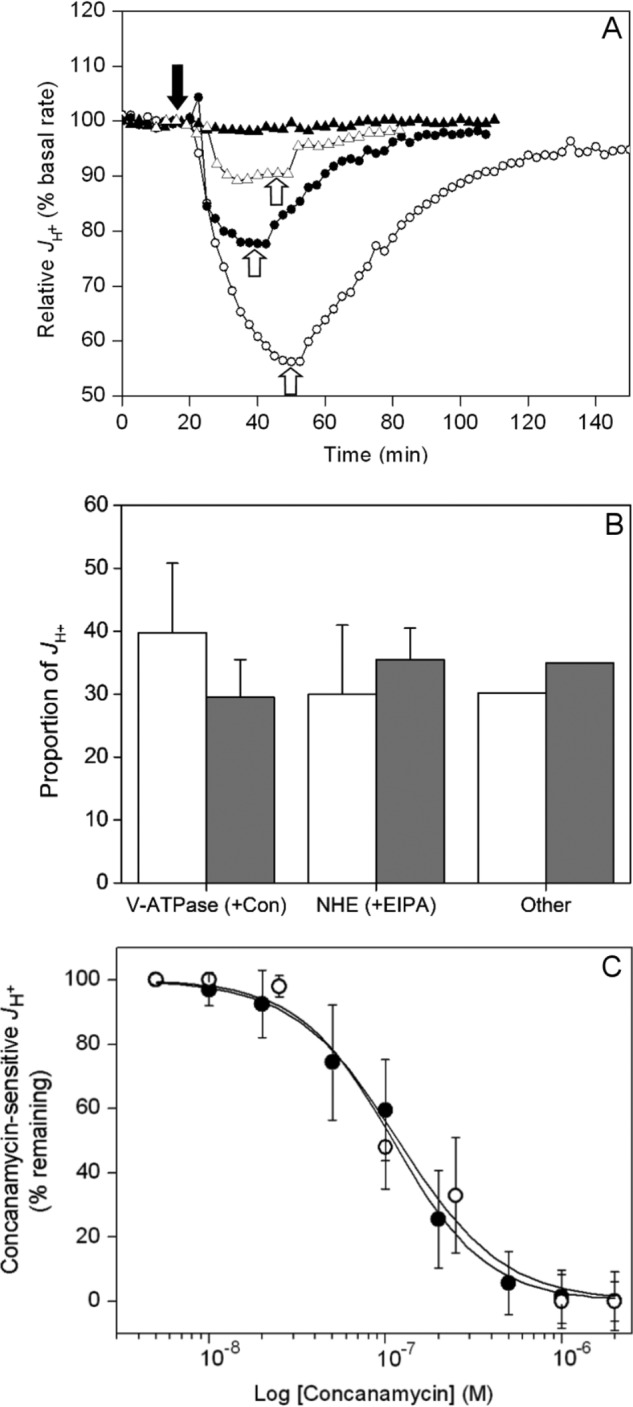
**V-ATPase activity at the plasma membrane of prostate carcinoma cells.** *A,* example microphysiometry traces for PC-3 cells. Concanamycin A was added as indicated by the *solid arrow* and washed out at the points indicated by the *open arrows. Solid triangles* indicate vehicle only (DMSO); *open triangles* indicate 50 nm; *solid circles* indicate 100 nm; *open circles* indicate 1 μm. *B,* relative contributions of V-ATPase and NHE1 activities to H^+^ extrusion by PC-3 (*white*, *n* = 10 (three readings per measurement)) and LNCaP (*gray, n* = 6 (3 cell samples per measurement)) prostatic carcinoma cells were determined as in *A* and expressed as the activity lost after addition of 2 μm concanamycin A (V-ATPase contribution), 50 μm ethyl isopropylamiloride (NHE1 activity), with the residual activity being the sum of other unassigned activities. *Error bars* represent the standard deviation for measurements with each inhibitor (PC-3, *n* = 10; LNCaP, *n* = 6). Differences between relative V-ATPase contributions to *J*_H_^+^ in the two cells types are not statistically significant (*p* = 0.055, unpaired *t* test). *C,* inhibition curves for the concanamycin-sensitive component of *J*_H_^+^ of PC-3 (*solid circles*) and LNCaP cells (*open circles*). Values are expressed as the percentage of the concanamycin-sensitive activity in *B* remaining after inhibitor addition. *Error bars* represent standard deviation of each measurement (*n* = 4). EC_50_ for both cells lines is ∼100 nm.

The contribution of the V-ATPase to proton extrusion activity across the plasma membrane of prostatic carcinoma cells (ECAR) was measured by microphysiometry after exposure to concanamycin. Two different PC-3 and LNCaP prostate cell lines were compared for correlation between V-ATPase activity and invasive phenotype. Mean basal rates of H^+^ pumping across the plasma membrane of PC-3 prostate cells gave a drop in extracellular pH of 0.32 pH units min^−1^, equivalent to a proton pumping rate (*J*_H_^+^) sufficient to change the proton concentration in the microphysiometer chamber by 43 nm min^−1^ (Table 1). Non-invasive LNCaP prostate cells displayed mean rates that were not significantly different from this (38 nm min^−1^; Table 1). Measurement after infusing with varying concentrations of concanamycin (Fig. 1*A*) caused a drop in proton extrusion rates (*t*_½_ ∼12 min), reversed by washing out the inhibitor (recovery *t*_½_ ∼40 min). For PC-3 cells, the maximal extent of inhibition by concanamycin indicates that 40 ± 14% of the basal *J*_H_^+^ is V-ATPase-dependent (Fig. 1*B*). We found a small but significant difference (*p* < 0.05) in the relative contribution of V-ATPase activity to *J*_H_^+^ of non-invasive LNCaP cells (29 ± 6%, Fig. 1*B*). Infusion with the Na^+^/H^+^ exchanger inhibitor ethyl isopropylamiloride at 100 μm blocked 30 and 34% of *J*_H_^+^ of PC-3 and LNCaP cells, respectively (Fig. 1*B*). Similar decreases in *J*_H_^+^ were observed when measurements were made with nominally sodium-free perfusion medium (Table 1). Although the apparent IC_50_ of 100 nm for concanamycin in both cells types (Fig. 1*C*) is high compared with published values for *in vitro* effects, such an elevated value is not unexpected for a cell-based assay and is below the concentration at which V-ATPase specificity is lost (52).

**TABLE 1:** **Rates of proton extrusion from prostatic carcinoma cells**

Cell type	Medium	ECAR	pH decrease*^a^*	*J*_H_^+^_, obs_
		μ*V s*^−*1*^	*units min*^−*1*^	*nm min*^−*1*^ *(*%*)*
PC-3	Na^+^-HBSS	320 ± 40*^b^* (*n* = 10)	0.32 ± 0.04	43.3 ± 5.4 (*100*)
PC-3	Choline-HBSS	211 ± 78 (*n* = 13)	0.21 ± 0.08	28.6 ± 10.6 (*66*)
LNCaP	Na^+^-HBSS	283 ± 61*^b^* (*n* = 9)	0.28 ± 0.06	37.9 ± 8.3 (*88*) (100) *^c^*
LNCaP	Choline-HBSS	184 ± 52 (*n* = 6)	0.18 ± 0.05	24.4 ± 7.0 (*56*) (64)*^c^*

*^a^* Data are from an initial pH of 7.4 (40 nm H^+^).

*^b^ t*_obt_ = 1.56, *p* > 0.05, not different (two-tailed t-test).

*^c^* Relative rates are for LNCaP cells.

**FIGURE 2.**
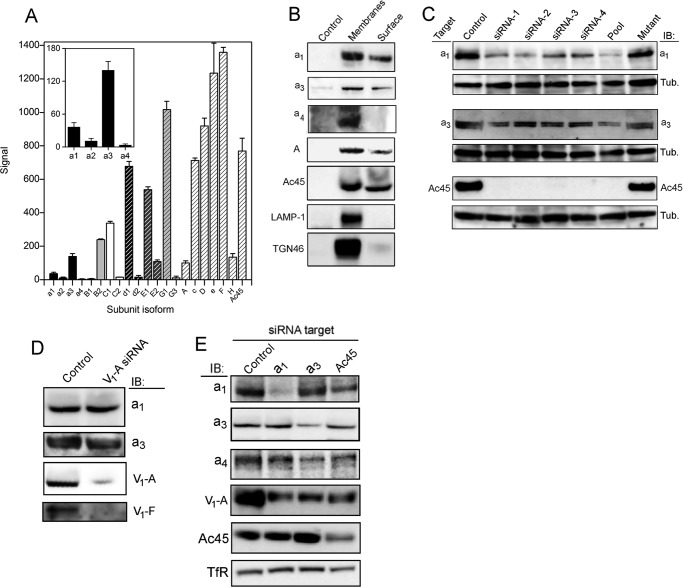
**Expression of V-ATPase subunit isoforms in PC-3 cells.** *A,* extracted mRNA from PC-3 cells was reverse-transcribed and analyzed on Affymetrix DNA microarrays (see “Experimental Procedures”). Mean values were determined from the output of two independent analyses. *Columns* indicating isoforms of a particular subunit have *equivalent shading*. Subunits represented on the array by only a single gene (A, c, D, e, F, H and Ac45) have the *same shading*. Output for the ATP6V0A1–4 genes is magnified in *inset. B,* to detect proteins accessible to the extracellular medium, intact PC-3 cells were labeled with membrane-impermeable biotinylation reagent prior to solubilization and extraction with StrepTactin affinity beads, followed by immunoblot analysis (*Surface*). As a control for reactivity, a detergent-solubilized membrane fraction was subjected to the same analysis (*Membranes*). Non-biotinylated protein gave negligible binding to the StrepTactin resin (*Control*). The Membranes samples contain 20 μg of total protein with the exception of the *a*_4_ blot, which contains 50 μg. Surface sample loading is the same in each blot, adjusted to give equivalent intensity in membranes and surface for Ac45. Chemiluminescence capture for *a*_4_ was 10-fold longer than for the other immunoblots. *C,* knockdown of *a*_1_, *a*_3_, and Ac45 expression by RNAi. PC-3 cell lysates (15 μg of protein) were analyzed by immunoblotting (*IB*) with the corresponding subunit-specific antibody after transfection with isoform-specific siRNAs. Tubulin (*Tub*) levels were used to ensure equivalent loading. Individual siRNAs that make up each Dharmacon SmartPool were individually tested (*siRNA1–4*), along with the corresponding pool (*Pool*). A version of siRNA-1 synthesized to include nucleotide changes as set out under “Experimental Procedures” was also tested (*Mutant*). *D,* RNAi knockdown of subunit A disrupts V_1_ assembly but does not affect subunit *a* expression. A total membrane fraction was isolated from cells after transfection with an siRNA pool specific for ATP6V1A. Lanes were loaded with 30 μg of total protein and immunoblotted (*IB*) for V_o_ subunits *a*_1_ and *a*_3_ and V_1_ subunits A and F. *E,* effects of siRNA knockdown on levels of non-targeted subunits at the plasma membrane. Cell surface proteins were extracted after biotinylation as in *B* from cells transfected with siRNAs for *a*_1_, *a*_3_, and Ac45. Biotinylated extract from 1 × 10^5^ cells (5 × 10^5^ for subunit *a*_4_) were analyzed by immunoblot (*IB*) for *a*_1/3/4_, subunit A, and Ac45. TfR was used as a loading control. Data capture for the *a*_4_ immunoblot was for 10× longer than for the other samples.

Having established that the V-ATPase is a major contributor to proton efflux across the plasma membrane of prostatic carcinoma cells, the relative levels of expression of subunit isoforms that could contribute to this activity were examined by gene array chip analysis (Fig. 2*A*). In PC-3 prostate cells, isoform B_2_ with subunit A predominates in the catalytic V_1_ domain, along with C_1_, E_1_, and G_1_ forms of the stator network subunits. Unexpectedly, the E_2_ isoform reported to be expressed exclusively in spermatids was also present at relatively high levels (Fig. 2*A*). Among V_o_ subunits, the *d*_1_ isoform was highly expressed along with the *a*_1_ and *a*_3_ forms of the large integral membrane component of the V-ATPase stator. The *a*_2_ isoform was expressed at low levels, and the *a*_4_ kidney-specific isoform was apparently absent. High levels of mRNA transcript encoding subunits D, F, H, *c,* and Ac45 were also detected (Fig. 2*A*). Note that although comparisons of levels of transcript for unrelated subunits are meaningless, comparisons between isoforms (presumed to have similar transcript length and stability) remain valid.

On the basis that *a*_1_ and *a*_3_ appeared to be the dominant membrane subunit isoforms, these were examined individually for their localization to the plasma membrane. To validate an assay for plasma membrane expression, surface biotinylation of PC-3 prostate cells with a membrane-impermeable cleavable biotinylation compound (sulfo-NHS-SS-biotin) was tested, followed by pulldown with StrepTactin beads. This generated a fraction that did not contain the late endosome/lysosome marker LAMP-1 and only very low levels of the Golgi marker TGN46 (Fig. 2*B*). Hence, we concluded that it represented exclusively a plasma membrane-enriched fraction. Only negligible binding of non-biotinylated proteins to the StrepTactin beads was observed. This fraction showed signal on immunoblots for both *a*_1_ and *a*_3_, along with V_1_ subunit A and Ac45 (Fig. 2*B*). Isoform *a*_1_ was detected with an antibody recognizing residues 71–210 (that has 32% identity with the corresponding part of *a*_3_) and *a*_3_ with an antibody raised against a peptide of residues 689–707 (11% identity to the corresponding *a*_1_ region) (53). Contrary to the expression data, the *a*_4_ isoform was also detectable in the total membrane fraction after long exposure of the chemiluminescent immunoblot. It was not detectable in the plasma membrane fraction when the protein loaded on the immunoblot was equivalent to that in the other lanes in Fig. 2*B*, but it was detectable at higher loading (discussed below). We were not able to detect the *a*_2_ isoform by immunoblot in either total membrane or plasma membrane fractions (data not shown). These data indicate that both *a*_1_ and *a*_3_ V-ATPase isoforms are simultaneously present at the plasma membrane of PC-3 cells.

To investigate isoform contributions to V-ATPase-dependent proton extrusion, we established the conditions needed for knockdown of V-ATPase subunit expression by RNA interference (RNAi) using synthetic inhibitory RNA (siRNA) oligonucleotides. Pools of siRNAs and their four constituent siRNAs were tested for effects on protein expression, along with a negative control version of one of the constituent siRNAs from each pool (see “Experimental Procedures”). Although relatively high siRNA concentrations (100 nm) and long transfection times (24 h) were required, ∼80% reduction in levels of *a*_1_ and *a*_3_ was achieved as judged by immunoblotting (Fig. 2*C*). Ac45 (ATP6AP1) has been implicated in V-ATPase accumulation at the plasma membrane of neuroendocrine cells (49) and in directing the V-ATPase to the ruffled membrane of activated osteoclasts, a specialized region of the plasma membrane (48). In PC-3 prostate cells, Ac45 is not only present at relatively high levels but also localized to the plasma membrane (Fig. 2*B*). To examine the role of Ac45 in V-ATPase trafficking, Ac45 was also subjected to RNAi knockdown, and the impact of this on different V-ATPase functions was measured. Treatment with 25 nm siRNA for 24–48 h gave >90% depletion of Ac45 levels (Fig. 2*C*). For *a*_1_, *a*_3_, and Ac45, treatment with a negative control mutant version of an siRNA from each pool resulted in no significant loss of expression (Fig. 2*C*), underlining the specificity of the expression knockdown. Depletion of subunit A expression by >90% was also achieved after 24 h with 25 nm of an siRNA pool (Fig. 2*D*).

Overall levels of *a*_1_ and *a*_3_ in the total membrane fraction were not affected by subunit A knockdown (Fig. 2*D*), indicating that V_o_ stability is not dependent on subunit A. Loss of subunit F however implies complete disruption of V_1_ assembly. In a mouse knock-out model, up-regulation of the B_2_ subunit can compensate for loss of B_1_ (54), with some functional compensation also possible between the subunit *a* homologues Vph1p and Stv1p in *Saccharomyces* (55). Here, knockdown of either *a*_1_ or *a*_3_ did not result in a corresponding increase in the levels of the other isoform at the plasma membrane (Fig. 2*E*), suggesting no compensatory mechanism. With 10-fold higher loading than in Fig. 2*B* and long exposure, some signal for the *a*_4_ isoform was also detected, but levels were similarly unaffected by knockdown of either *a*_1_ or *a*_3_. Depletion of these subunit isoforms did result in a decrease in the signal from subunit A associated with the plasma membrane fraction, consistent with decreased assembly due to reduced availability of V_o_. Ac45 knockdown had a similar effect on subunit A and *a*_1_ levels (but no reproducible effect on *a*_3_) (Fig. 2*E*), consistent with a role for the accessory subunit in V-ATPase delivery to the cell surface.

##### Intracellular Localization of V-ATPase Subunit Isoforms

**FIGURE 3.**
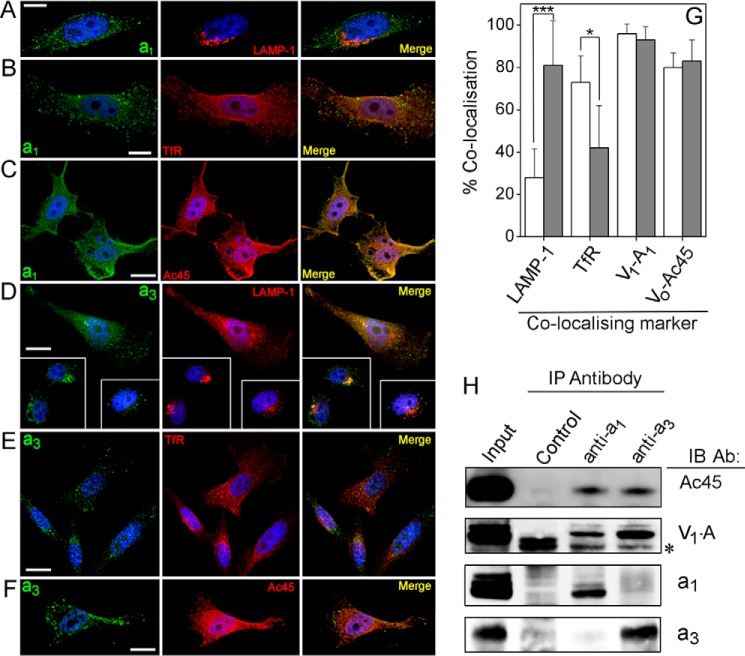
**Subcellular localization of the *a*_1_ and *a*_3_ subunits.** *A–F,* immunofluorescence microscopy of fixed and permeabilized PC-3 cells. *Left column,* cells stained with antibodies to *a*_1_ (*A–C*) or *a*_3_ (*D–F*). *Center column,* staining with antibodies to LAMP-1 (*A* and *D*), TfR (*B* and *E*), or Ac45 (*C* and *F*). *Right column,* merged images of those in *left* and *center columns*, with coincident pixels colored *yellow*. Nuclei are stained with Hoechst 33258. *Scale bars,* 10 μm (*A* and *B*) or 20 μm (*C–F*). *G,* quantitation of co-incident staining. Images such as those in *A–F* were analyzed using the Coincidence function of Imaris software. Values are expressed as the percentage of pixels showing staining for *a*_1_ (*open histograms*) or *a*_3_ (*gray histograms*) that also stain for the subcellular marker indicated. *Error bars* represent standard deviation. The *a*_3_ isoform showed significantly greater (*p* < 0.01) co-localization with the late endosomal marker LAMP-1 (38 cells analyzed) than did *a*_1_ (26 cells analyzed), *a*_1_ co-localizing significantly more than *a*_3_ (*p* < 0.05) with the plasma membrane/endosome marker TfR (23 and 27 cells, respectively). Both *a*_1_ and *a*_3_ showed strong co-localization with subunit A and Ac45 (11–24 cells analyzed). *, *p* < 0.01; ***, *p* < 0.001. *H,* both *a*_1_ and *a*_3_ associate with Ac45. The *a*_1_ and *a*_3_ subunit isoforms were immunoprecipitated (*IP*) from detergent-solubilized PC-3 cell lysate, and co-precipitating proteins were analyzed by immunoblotting (*IB*). Precipitation with normal rabbit serum was used as a control for nonspecific binding. A sample containing the equivalent amount of cell lysate to that used in the immunoprecipitations was also run (*Input*). In the immunoblot of the A subunit, the *asterisk* indicates recognition of the heavy chain of the precipitating IgG. *Ab,* antibody.

An investigation of the intracellular distribution of the V-ATPase *a*_1_ and *a*_3_ subunits by immunofluorescence microscopy revealed distribution in different (but in part overlapping) compartments (Fig. 3). Staining for the *a*_1_ isoform was punctate (consistent with its presence in small vesicles) and distributed throughout the cytoplasm (Fig. 3, *A–C*). This staining was coincident with that of TfR (CD71), a marker for the plasma membrane and early endosomes (Fig. 3*B*), but not with the late endosome/lysosome marker LAMP-1 (Fig. 3*A*). The converse situation was observed with the *a*_3_ isoform; it showed much greater coincidence with LAMP-1 than with TfR (Fig. 3, *D* and *E*). Both *a*_1_ and *a*_3_ showed substantial co-distribution with Ac45 and A (Fig. 3, *C* and *F*). Quantification of pixel co-distribution in merged optical sections (Fig. 3, *A–F*) confirmed that *a*_1_ predominantly co-localizes with TfR-enriched early endosomes, whereas *a*_3_ is predominantly associated with LAMP-1-containing late endosomes/lysosomes (Fig. 3*G*). Both *a*_1_ and *a*_3_ showed ∼95% co-distribution with A, suggesting relatively little V_o_ detached from V_1_, and ∼80% co-distribution with Ac45, suggesting that the majority of V-ATPases with either subunit *a* isoform associates with this accessory subunit. On the basis of these data, it appears that *a*_1_ and *a*_3_ exist largely in different compartments of the endocytotic machinery, albeit with some degree of overlap. Immunoprecipitation of detergent-solubilized V-ATPase complexes (Fig. 3*H*) showed that antibodies to either *a*_1_ or *a*_3_ isoforms co-precipitated Ac45 and A subunits. Precipitates generated by pulldown with either anti-*a* antibody gave no signal on immunoblots probed with the antibody to the other isoform (Fig. 3*H*), confirming that the respective antibody probes were highly isoform-specific.

##### Functional Effects of RNAi Knockdown of V-ATPase Subunits

**FIGURE 4.**
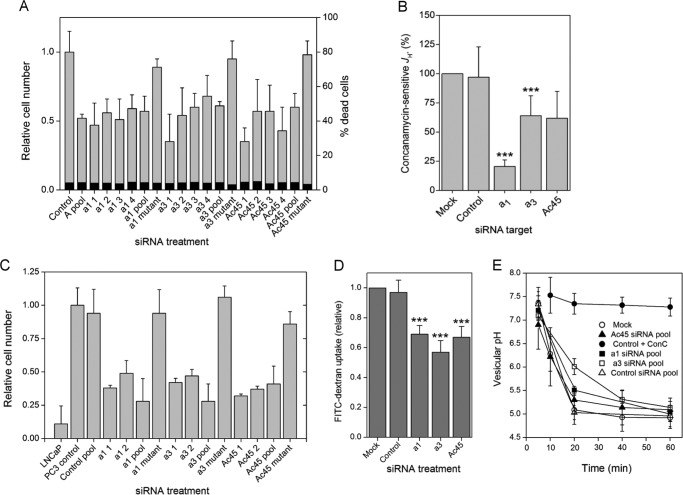
**Effects of siRNA knockdown on V-ATPase function in PC-3 cells.** *A,* assay of live/dead cells after siRNA treatment. As in Fig. 1, individual siRNAs that make up each pool were individually tested (*siRNA1–4*), along with the corresponding pooled siRNAs and mutant version. Numbers of viable cells 48 h after siRNA transfection were measured by calcein fluorescence and expressed relative to the change in number of cells in a control transfected with non-targeting siRNA. Numbers of dead cells (as a percentage of the total) were measured by propidium iodide uptake (measurements made in duplicate or triplicate in 96-well format, repeated 4 times). *B,* effect of non-targeting (*control*) and *a*_1_, *a*_3_, and Ac45-specific siRNAs on concanamycin-sensitive H^+^ efflux. The concanamycin-sensitive activity remaining after siRNA treatment was determined by adding the compound (2 μm) and expressed relative to the activity in control cells (equivalent to the concanamycin-sensitive activity shown in Fig. 1*B*). *Error bars* indicate standard deviation of each measurement (*n* = 3, two cell samples per measurement). Both *a*_1_ and *a*_3_ siRNAs significantly decreased the concanamycin-sensitive component of *J*_H_^+^ (*a*_1_, *p* < 0.01; *a*_3_, *p* < 0.05). *C, a*_1_, *a*_3_, and Ac45 siRNAs suppress the invasive phenotype of PC-3 cells. PC-3 cells were induced to migrate through Matrigel using FBS as chemoattractant. Cells treated with siRNAs (two individual siRNAs from each pool, the complete four siRNA pool and a mutant version (detailed under “Experimental Procedures”)) were used 24 h post-transfection. Cells migrating through the Matrigel layer were counted after 24 h, and numbers were expressed relative to the untreated PC-3 cell control. Invasiveness of mock-transfected cells (*Control pool*) was not significantly different from untreated cells. PC-3 cells were significantly more invasive than a non-invasive LNCaP control (*p* < 0.001), and *a*_1_, *a*_3_, and Ac45 siRNAs (individual or pooled) all showed significantly decreased cell invasiveness (*p* < 0.01; *n* = 3, with three replicates per determination). *D* and *E,* uptake of FITC-dextran and measurement of vesicular pH. *D*, total uptake after 10 min measured as the fluorescence emission at 520 nm for cells treated with siRNAs that were non-targeting pool (*Control*) or pools targeted to Ac45, *a*_1_, or *a*_3_ subunits. *Error bars* show standard deviation (*n* = 3). All targeting siRNAs caused significant effects (***, *p* < 0.001). *E,* change in the vesicular pH experienced by the endocytosed FITC-dextran, measured as the change in the 492 nm/442 nm excitation ratio for emission at 520 nm. *Open circles,* untreated cells; *closed circles,* 2 μm concanamycin; *open triangles,* control (non-targeting) siRNA; *closed triangles,* Ac45 siRNA. *Closed* and *open squares, a*_1_ and *a*_3_ siRNAs, respectively. *Error bars* indicate standard deviation (*n* = 3).

Although PC-3 cells transfected with a non-targeting control siRNA pool and with mutant versions of individual siRNAs proliferated at essentially normal rates (doubling time of ∼48 h), cultures of cells depleted for V-ATPase subunits were essentially static (Fig. 4*A*). The transfected cells did however remain alive (as judged by calcein-AM uptake), with no appreciable differences in the proportion of dead cells between control and V-ATPase-depleted cultures (4–5%; Fig. 4*A*). Cessation of growth after V-ATPase knockdown serves to underline the importance of its function in cell physiology.

The effects of RNAi knockdown of *a*_1_/*a*_3_ levels on the invasive properties of PC-3 cells were also tested by measuring chemotactic migration through an extracellular matrix extract (Matrigel) (Fig. 4*C*). Knockdown of either *a*_1_ or *a*_3_ by individual or pooled siRNAs strongly suppressed migration in this model of metastatic potential, clearly indicating that both isoforms make significant contributions to the invasive phenotype of PC-3 cells. Mutations to inhibitory siRNAs from both *a*_1_ and *a*_3_ pools that permitted normal levels of expression also led to cells achieving normal levels of invasiveness (Fig. 4*C*), further indicating the specificity of the RNAi effect.

Loss of invasiveness of breast cancer cells after *a*_3_ knockdown has previously been reported, with effects similar to those caused by treatment with bafilomycin A1 (33). It is unclear whether these effects are mediated via a decrease specifically in *J*_H_^+^ or through broader cellular effects such as arrest of trafficking. Pertaining to this, we have observed that the cytokine receptor activator of nuclear factor κB ligand has no effect on *J*_H_^+^, but the ∼50% increase in invasiveness it causes is completely ablated by concanamycin A (data not shown). Receptor activator of nuclear factor κB ligand is reported to increase invasiveness of prostate and breast cancer cells of epithelial origin via c-Src and MAPK signaling pathways (56, 57). As discussed below, knockdown of *a*_3_ in particular affects invasion to a much greater extent than it does *J*_H_^+^ (Fig. 4*B*). Consequently, it is likely that the effects of *a*_1_ and *a*_3_ knockdown are mediated via changes to trafficking processes in the cell rather than to decreased acidification of the extracellular environment.

Ac45 depletion by siRNA pool and by individual RNAs also significantly decreased PC-3 cell invasiveness *in vitro*, although invasiveness was unaffected by a mutant version of representative siRNA (Fig. 4*C*). This points to a role for Ac45 in navigating both V-ATPase isoforms around the cell, a phenomenon we investigated further in this study.

Knockdown of *a*_1_ levels eliminated 78 ± 6% of the concanamycin-sensitive component of *J*_H_^+^, whereas depletion of *a*_3_ levels accounted for only 34 ± 17% (Fig. 4*B*), indicating that it is predominantly an *a*_1_-containing V-ATPase that contributes to *J*_H_^+^ in these cells, with an *a*_3_ isoform making a much smaller (but significant) contribution to this activity. Because efficiency of RNAi knockdown of each isoform is broadly equivalent (Fig. 2*C*), the data in Fig. 4*B* suggest an ∼2:1 split in the contribution of the two isoforms to the bafilomycin-sensitive *J*_H_^+^ component. We note however that the sum of effects of even incomplete *a*_1_ or *a*_3_ knockdown on plasma membrane V-ATPase activity is >100% inhibition, implying that *a*_3_ knockdown has some influence on plasma membrane activity of the *a*_1_-containing isoform and vice versa. This is not unexpected given the dynamic inter-relationship between membrane compartments within the cell. Depletion of Ac45 also caused a trend toward loss of concanamycin-sensitive *J*_H_^+^, but these measurements were prone to high variance and hence the trend indicating 40% loss of activity was not significant at the 95% confidence limit (Fig. 4*B*).

Fluid-phase endocytosis was significantly reduced after Ac45, *a*_1_, or *a*_3_ knockdown as indicated by a decreased uptake of FITC-labeled dextran (Fig. 4*D*). However, neither the rate of endosomal/lysosomal vesicular acidification nor the final minimum pH levels experienced by the dextran in vesicles were significantly different from controls when *a*_1_ or Ac45 was depleted by siRNA transfection (Fig. 4*E*). Only *a*_3_ knockdown appeared to have any effect on this process, initially slowing the rate at which the pH of FITC-dextran-loaded vesicles decreased, but ultimately with no significant effect on the minimal pH achieved.

##### Requirement for V-ATPase Subunits in Endocytosis and Trafficking

**FIGURE 5.**
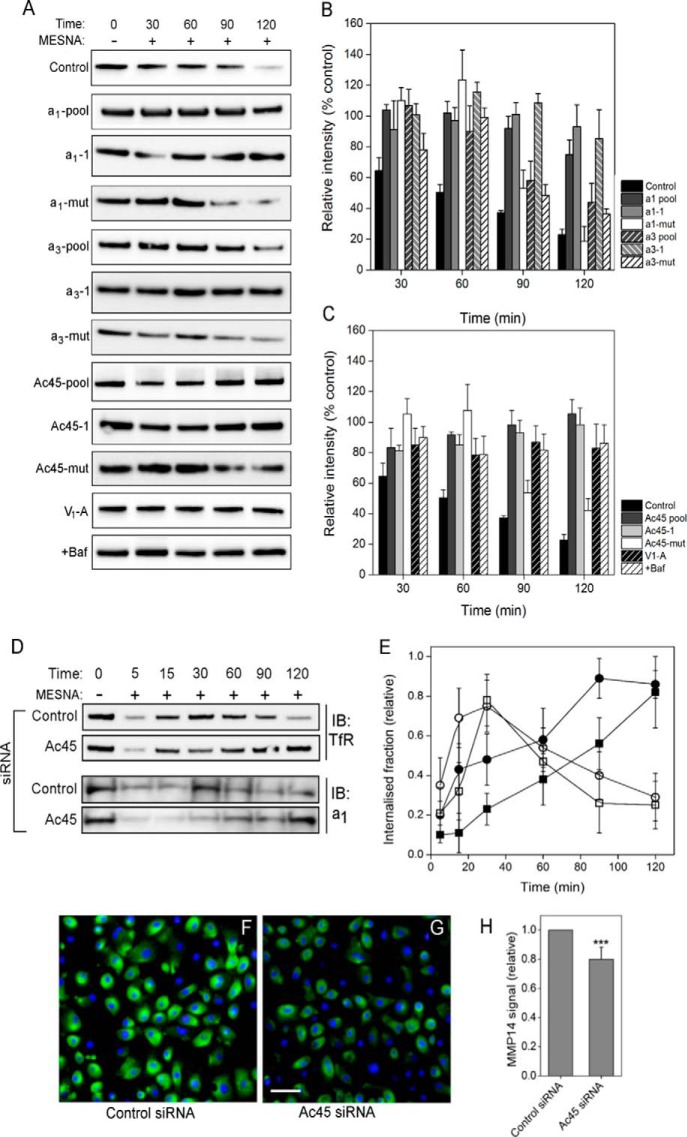
**Effects of subunit *a*_1_, *a*_3_, and Ac45 depletion on TfR recycling dynamics.** *A,* recycling of the TfR was monitored using the surface biotinylation assay as under “Experimental Procedures” in cells treated with non-targeting siRNA pool (*Control*); siRNA pools targeted to *a*_1_, *a*_3_, Ac45 (*a*_1_/*a*_3_/*Ac45-pool*); representative individual siRNAs from each pool (*a*_1_/*a*_3_/*Ac45-1*); and the corresponding mutant version of that siRNA (*a*_1_/*a*_3_/*Ac45-mut*). To monitor the effects of disruption of proton pumping, cells were treated with an siRNA pool targeted to subunit A (*V1-A*) or pre-treated for 30 min with 100 nm bafilomycin A1 (+*Baf*). After stripping residual surface biotinylation with MESNa, internalized biotinylated proteins were extracted from solubilized cells by binding to StrepTactin beads. Internalized TfR retaining biotin was detected by immunoblotting. *B* and *C,* quantitation of immunoblots as in *A* by densitometry. Values are expressed as a proportion (%) of the maximally biotinylated state at time 0. *Error bars* indicate standard deviation of the mean value (*Control, n* = 3). *D,* Ac45 depletion arrests recycling of plasma membrane proteins. Recycling of the transferrin receptor (*TfR*) and the *a*_1_ isoform was monitored after siRNA knockdown of Ac45 using a surface biotinylation assay as above. At the specified time points, biotinylated proteins were extracted by binding to StrepTactin beads. Bound proteins were analyzed by immunoblotting, typical examples being shown for transferrin receptor (*D, upper panel*) and *a*_1_ (*D, lower panel*). *E,* quantitation of immunoblots by densitometry. *Open symbols,* cells treated with control (non-targeting) siRNA. *Closed symbols,* treated with Ac45 siRNA. Values are expressed as a proportion of the maximally biotinylated state at time 0. *Error bars* indicate standard deviation (TfR, *n* = 5; *a*_1_, *n* = 3). *F–H,* Ac45 depletion decreases cell surface expression of MMP-14. Sample immunofluorescence images of non-permeabilized control (*F*) or Ac45 siRNA-transfected (*G*) PC-3 cells labeled with an antibody recognizing the extracellular domain of MMP-14. Nuclei are stained with Hoechst 33258. *Scale bar,* 20 μm. *H,* quantitation of the labeling in control and Ac45-depleted cells. Fluorescence from raw images (15 random fields containing ∼100 cells from three separate experiments) was quantified using ImageJ. ***, *p* < 0.001.

Substantial co-distribution of *a*_1_ with TfR implies that they move together through the endosomal recycling system. To examine this, we tested the impact of siRNA depletion of *a*_1_ and *a*_3_ on TfR (CD71) recycling using a surface biotinylation protection assay. This involves membrane protein biotinylation (on ice) of extracellular surface-exposed amine groups with a membrane-impermeable but reversible (disulfide-containing) reagent, followed by resumption of endocytosis by incubation at 37 °C. Biotinylated membrane protein internalization prevents removal of the biotin moiety by incubation with a water-soluble reductant (MESNa); hence, pulldown of the total biotinylated fraction from cell lysates reports on the kinetics of endocytosis. For control cells, biotin removal indicative of TfR re-exposure at the cell surface occurred with a half-time of ∼45 min (Fig. 5, *A* and *B*). Transfection with siRNAs targeted against the *a*_1_, *a*_3_, Ac45 (pooled or individual siRNAs), or A soluble domain subunits had no significant effect on the initial internalization, with broadly equivalent levels of internalized TfR consistently seen up to 30 min after restart of endocytosis (Fig. 5, *A–C*). However, knockdown of *a*_1_ and Ac45 significantly slowed the rate of biotin removal from TfR, with levels of receptor biotinylation remaining essentially constant across the 120-min time course, indicating retention of the receptor inside the cell. Treatment with an *a*_3_-depleting siRNA pool caused some slowing of TfR recycling, an individual siRNA from that pool actually having a more clearly defined effect (Fig. 5, *A* and *B*). Depletion of *a*_1_ and Ac45 (and less conclusively *a*_3_) therefore significantly slowed transit of TfR through the endosomal recycling system. Because of the non-catalytic role of V_o_ in membrane fusion (58, 59), the observed effect could be due either to loss of acidification or to inhibition of membrane fusion events. Because membrane fusion should not be affected by loss of V-ATPase catalytic activity, effects resulting from RNAi knockdown of the catalytic subunit A can discriminate between these possible mechanisms. Subunit A depletion (Fig. 5*A*) stalled TfR recycling in a similar way to *a*_1_ knockdown without affecting levels of *a*_1_ in the membrane (Fig. 2*D*), and it was indistinguishable from the effect of directly inhibiting proton pumping with bafilomycin A1 (Fig. 5, *A* and *C*). Arrest of TfR recycling by *a*_1_ depletion is likely to be mediated via altered vesicular acidification and not by direct effects on membrane fusion. The siRNA effects appeared to be highly specific, with mutant versions of expression-suppressing siRNAs having no significant effects on TfR recycling.

The impact of Ac45 depletion on plasma membrane protein recycling was further resolved using the surface biotinylation assay. In control cells, TfR was internalized with a *t*_½_ of ∼15 min (Fig. 5, *D* and *E*), with loss of biotinylation after ∼2 h consistent with recycling back to the cell surface (or degradation). Knockdown of Ac45 elicited a major change in uptake kinetics; TfR internalization was slowed, with a *t*_½_ of ∼45 min; recycling of TfR back to the plasma membrane could not be detected even after 2 h (Fig. 5, *D* and *E*). A similar change in the dynamics of subunit *a*_1_ internalization and recycling was also observed (Fig. 5, *D* and *E*). Maximal *a*_1_ internalization in Ac45-depleted cells occurred after >2 h compared with ∼30 min in control cells. These data indicate that the *a*_1_-containing V-ATPase, although active at the plasma membrane, is endocytosed relatively quickly in an Ac45-dependent manner.

The observed effect of Ac45 depletion on membrane cycling to the cell surface could also impact the delivery/secretion of ECM-degrading enzymes, offering an explanation for the impact of Ac45 knockdown on PC-3 cell invasiveness *in vitro* (Fig. 4*C*). To examine this further, we looked at the effect of Ac45 depletion on delivery of the membrane-bound matrix metalloprotease MMP-14 to the cell surface. Highly expressed at the plasma membrane of cancer cells (60), MMP-14 activates collagenases leading to degradation of the ECM and is specifically linked to cell invasiveness (61, 62). Here, using immunofluorescence labeling of non-permeabilized cells with an antibody recognizing the extracellular region of MMP-14 (Fig. 5, *F* and *G*), Ac45 depletion caused a significant decrease in antibody-accessible protein at the cell surface (Fig. 5*H*). Disturbance of V-ATPase dynamics through loss of Ac45 therefore not only impacts the ability of the cell to sustain a low extracellular pH (Fig. 4*B*) but also its ability to secrete ECM degrading activities. Both are important factors in expression of the invasive phenotype.

**FIGURE 6.**
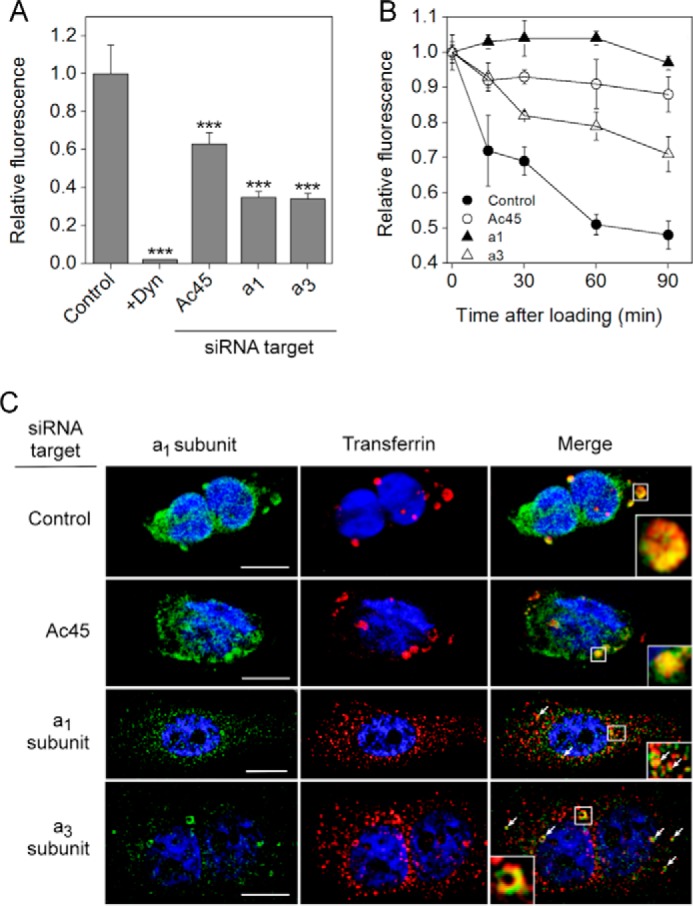
**Effects of Ac45 expression knockdown on endocytosis of transferrin.** *A,* Alexa-594-Tf uptake by PC-3 cells treated with control (non-targeting), Ac45, *a*_1_- or *a*_3_-targeted siRNA pools. As negative control, cells were also treated with the endocytosis inhibitor dynasore (*Dyn*). After washing off excess transferrin, cells were fixed for microscopy, and the fluorescence from raw images was determined (10–25 images) using ImageJ software. All treatments resulted in significant (*p* < 0.01) loss of transferrin uptake. ***, *p* < 0.01. *B,* time-dependent changes in Alexa-594-Tf fluorescence in siRNA treated cells. After the initial transferrin loading, cells were returned to 37 °C before fixing at different time points. Alexa-594-Tf fluorescence was determined as in *A. Closed circles,* control (non-targeting) siRNA pool; *open circles,* Ac45 siRNA pool; *closed triangles, a*_1_ siRNA pool; *open triangles, a*_3_ siRNA pool. On average, 20 images were captured and quantified for each time point for each siRNA treatment. *C,* immunofluorescence microscopy of fixed and permeabilized siRNA-treated cells 90 min after initiation of Alexa-594-Tf uptake, stained for the *a*_1_ subunit (FITC, *left column*). *Center column*, Alexa-594-Tf fluorescence. *Right column,* merged images with coincident pixels colored *yellow. Insets* and *arrows*, details of vesicular structures showing coincident fluorescence. Contrast has been adjusted to highlight qualitative aspects, but quantitation was performed on raw images captured using constant microscope parameters. Nuclei are stained with Hoechst 33258. *Scale bar,* 20 μm.

The effects of V-ATPase subunit depletion on transit through the endosome-lysosome system were also monitored by following time-dependent changes in the intracellular distribution and total fluorescence signal of Alexa-594-Tf as a proxy for its receptor, TfR (Fig. 6). After initial binding of labeled Tf ligand for 30 min at 4 °C, cells showed characteristic labeling of the cell periphery that was qualitatively similar regardless of siRNA applied. Quantitatively, Ac45 knockdown decreased Tf uptake by ∼40% (Fig. 6*A*), with an even greater effect observed upon knockdown of *a*_1_ (65% decrease) or *a*_3_ (64% decrease). Treatment of control cells with the pharmacological dynamin inhibitor “dynasore” (63) almost completely abolished Tf endocytosis (Fig. 6*A*). Monitoring Alexa-594-Tf fluorescence over time allowed Tf recycling and release from the cell to be monitored (Fig. 6*B*). In control cells, ∼54% of the signal was lost over a 90-min period. However, knockdown of Ac45 or *a*_1_ levels caused the Alexa-594-Tf signal to decrease by no more than 12% over this period, consistent with retention of the labeled Tf in the endosomal system. Depletion of *a*_3_ in the cell also appeared to slow Tf recycling, although this effect was not as pronounced as in Ac45/*a*_1_-depleted cells. In control cells, internalized transferrin co-localized with *a*_1_ in large perinuclear vesicular bodies (Fig. 6*C*, *row 1*), qualitatively similar to previous observations (64) and presumed to be recycling endosomes. Cells in which Ac45 was depleted showed qualitatively similar distribution of large Tf/*a*_1_-containing vesicles (Fig. 6*C*, *row 2*). This infers that although transferrin uptake is decreased as a result of Ac45 depletion (as shown in Fig. 6*A*) because TfR is retained in the cell (Fig. 5, *D* and *E*), the endocytosed Tf ultimately ends up in the same compartment as in control cells. The key difference is that in the absence of Ac45, the large vesicular bodies in which Tf accumulates recycle only very slowly back to the cell surface. Hence, the effects of Ac45 knockdown appear to inhibit both internalization from the plasma membrane and transit back to the cell surface.

Although the effects of depleting either subunit *a* isoform showed broadly similar effects to Ac45 depletion by inhibiting Tf endocytosis (Fig. 6*A*) and accumulation within intracellular compartments (Fig. 6*B*), there were marked differences in the intracellular distribution of internalized Tf. After 90 min, Tf was still distributed throughout the *a*_1_-depleted cells in small vesicular bodies, with partial co-localization with the residual *a*_1_ subunit (Fig. 6*C*, *row 3, arrows* and *inset*). A similar distribution pattern was seen after *a*_3_ knockdown (Fig. 6*C*, *row 4*), with some evidence for the presence of larger *a*_1_-containing vesicles (*inset* and *arrows*). This was in contrast to the large perinuclear vesicles evident in both control and Ac45-depleted cells, suggesting that subunit *a*_1/3_ and Ac45 depletion exert their effects at different stages in the endosomal transport process.

## Discussion

In this study, invasive and non-invasive prostate carcinoma cells supported high but essentially indistinguishable overall rates of proton extrusion. The ∼36% higher V-ATPase-dependent component of this activity from invasive cells above that from non-invasive was statistically significant, but this difference appears too small to be a critical factor in defining the invasive phenotype. In contrast to previous studies (31, 33), we conclude that in the prostate carcinoma cell lines examined here, V-ATPase function at the plasma membrane differs little between invasive and non-invasive cells. Consequently, we detect no direct correlation between plasma membrane V-ATPase activity and metastatic potential. This does not mean that V-ATPase activity has no role in invasiveness as it clearly does, given that inhibition by either bafilomycin or RNAi has a significant impact on invasiveness (31, 33). Rather than being a feature that distinguishes invasive from non-invasive cells, we suggest that plasma membrane V-ATPase facilitates invasiveness by supporting ECM degradation via extracellular acidification or transport/secretion of protease cargo (32, 65–67). This latter role is consistent with the observation that Ac45 knockdown decreases invasiveness with only a small loss of *J*_H_^+^, presumably as a consequence of stalling transport processes (discussed below).

The microscopy data and RNAi effects on TfR recycling indicate that the *a*_1_ and *a*_3_ isoforms primarily operate in different endosome populations. The distribution of the *a*_1_ isoform within the cell strongly resembles that of TfR (Fig. 3), suggesting that it also transits through early endosomes and recycles back to the plasma membrane (68, 69). Bafilomycin brings about rapid alkalinization of compartments along the endosome-lysosome route but has only a small effect on the rate of TfR internalization from the plasma membrane (70) or trafficking of cargo from the sorting endosome to the endosomal recycling compartment (ERC) (71, 72). In contrast, bafilomycin significantly slows exit of cargo from the ERC (72), suggesting that it is this stage in the recycling process that is most affected by loss of V-ATPase activity after RNAi depletion of *a*_1_. The *a*_3_ form largely populates late endosomes/lysosomes, yet it also traffics to the plasma membrane. This isoform is recruited from late endosomes/lysosomes to plasma membrane-derived structures in macrophages and in osteoclasts via interaction with microfilaments (14, 18, 73). A general role in secretory pathways has been proposed for this isoform (24) that could extend to the prostatic epithelial cells examined here, where it could be present on secretory lysosomes. It is noteworthy that both *a*_1_ and *a*_3_ knockdown decrease Tf internalization, slowing its transit through the endosome network. Depletion of either *a* subunit also leads to significant differences in morphology and intracellular distribution of TfR/*a*_1_ vesicles compared with control cells (and to Ac45-depleted cells, discussed below) visualized by antibody staining or chase of fluorescent Tf ligand through the transport pathways. Complete and efficient trafficking of the Tf-TfR complex through the endosomal system must therefore require both *a*_1_ and *a*_3_ forms of the V-ATPase. Given that subunit A knockdown leads to a slowing of TfR recycling similar to that seen with *a*_1_ (and to a lesser extent *a*_3_), it seems most plausible that this retention effect results from loss of V-ATPase activity. Of course this does not exclude the possibility that loss of V_o_ resulting from *a*_1_ (or *a*_3_) knockdown also prevents a final (ATPase-independent) membrane fusion process at the plasma membrane perhaps involving a connection to the R-SNARE.

In the degradative pathway, the movement of cargo from the late endosome to lysosome (but not entry into the late endosome) is also dependent on V-ATPase activity (71, 74). Here, knockdown of either *a*_1_ or *a*_3_ isoforms (also Ac45) had an effect on this process, inhibiting the overall level of dextran uptake into the cell. However, because this FITC-dextran uptake was inhibited without any apparent alkalinization of the late endosomes/lysosome, we conclude that knockdown of the V-ATPase must lead to overall loss of cellular capacity for fluid-phase endocytosis and transition to the lysosome but with the residual V-ATPase expression still sufficient to achieve normal luminal pH.

Recruitment of proteins to endosomal membranes is pH-dependent (75, 76), and hence V-ATPase proton-pumping capacity is important in defining the function of the compartment. Here, *a*_1_ and *a*_3_ isoforms were differentially targeted to cellular compartments that have different luminal pH (>6 for *a*_1_ in early endosomes/ERC and <6 for *a*_3_ in late endosomes/lysosomes). An intriguing possibility is that the presence of *a*_1_ or *a*_3_ programs the V-ATPase to maintain a particular luminal pH. This could result from perhaps different ATP/H^+^ coupling ratios (77) or via a pH-sensing regulatory feedback system with similarities to that proposed for the *a*_2_ subunit isoform (78).

The V_o_-associated glycoprotein Ac45 (ATP6AP1 and ATP6S1) plays a major role in navigating the V-ATPase around prostate carcinoma cells, as it does in the secretory pathway of neuroendocrine cells (49). The question is as follows. Does Ac45 facilitate transport to the plasma membrane, internalization from the plasma membrane, or both stages in the transport process? Ac45 does function in clathrin-coated vesicle formation and clathrin-mediated endocytosis (79), with internalization from the plasma membrane mediated via recognition sequences in its 26-residue cytoplasmic tail (80). Consistent with a function in guiding the V-ATPase to the plasma membrane, mutations in this same cytoplasmic tail lead to failure to recruit *a*_3_-containing V-ATPase to the ruffled membrane of osteoclasts (48). Ac45 transgene expression has furthermore been shown to cause V-ATPase accumulation at the plasma membrane (49). Consequently, roles in transport both to and from the plasma membrane are supported, albeit in different cell types. This is reiterated here in invasive prostate carcinoma cells, although Ac45 depletion appears to have a predominant effect on transit to the plasma membrane. The partial loss of plasma membrane V-ATPase activity in Ac45-depleted cells indicates a net decrease in V-ATPase transport to the plasma membrane, although we have been unable to determine whether just *a*_1_ or both *a*_1_ and *a*_3_ isoforms are affected. However, based on their shared contribution to the overall *J*_H_^+^ in PC-3 cells, both are likely to be affected to some extent. Most significantly, Ac45 knockdown also arrests recycling of Tf and TfR. During chase experiments, the morphology and organization of transferrin-loaded vesicles in Ac45-depleted cells was very similar to that in control cells, suggesting that Tf-TfR was retained at a relatively late stage in the recycling process by failing to exit the ERC or linked to a function of Ac45 in late-stage exocytosis (50). Incomplete knockdown of *a*_1_ or *a*_3_ had similar effects to Ac45 depletion on recycling of TfR, but the differences in vesicle morphology resulting from these knockdown experiments implies that they affect the process at a different earlier stage. Ac45 depletion also had some effect on the kinetics of internalization of TfR and the *a*_1_ isoform, slowing their removal from the plasma membrane, again consistent with the reported effects of V-ATPase subunit depletion on endocytosis (79). We therefore conclude that although Ac45 is an important factor in navigating the V-ATPase through the endosomal system, the effect of its knockdown is not simply due to a failure to bring the acidifying enzyme to the correct compartment. Instead, its role is in targeting the V-ATPase to the correct place in the late stages of membrane fusion during exocytosis. If Ac45 is a significant factor in navigating the V-ATPase to the plasma membrane, enhanced expression could be responsible for maintaining the high levels of surface V-ATPase activity in cancer cells.

## Author Contributions

M. A. H., S. P. M., and S. P. conceived and planned the study. G. A. S., M. A. H., and C. P. collected data that was analyzed by M. A. H., G. A. S., S. P. M., and S. P. G. J. H. performed fluorescence microscopy and assisted with computational analysis of images. M. A. H., S. P. M., and S. P. prepared the manuscript. All authors reviewed the data and approved the final version of the manuscript.

## Footnotes

6 The abbreviations used are:

ECM: extracellular matrix
ECAR: extracellular acidification rate
ERC: endosomal recycling complex
HBSS: Hanks' basal salt solution
TfR: transferrin receptor
V-ATPase: vacuolar H^+^-ATPase
Tf: transferrin.
